# Functional Characterization of the *spf/ash* Splicing Variation in OTC Deficiency of Mice and Man

**DOI:** 10.1371/journal.pone.0122966

**Published:** 2015-04-08

**Authors:** Ana Rivera-Barahona, Rocío Sánchez-Alcudia, Hiu Man Viecelli, Veronique Rüfenacht, Belén Pérez, Magdalena Ugarte, Johannes Häberle, Beat Thöny, Lourdes Ruiz Desviat

**Affiliations:** 1 Centro de Biología Molecular Severo Ochoa, Universidad Autónoma de Madrid-Consejo Superior de Investigaciones Científicas, Madrid, Spain; 2 Centro de Investigación Biomédica en Red de Enfermedades Raras (CIBERER), Madrid, Spain; 3 Instituto de Investigación Biomédica Hospital La Paz (IdiPAZ), Madrid, Spain; 4 Division of Metabolism and Children’s Research Centre (CRC), University Children’s Hospital, Zürich, Switzerland; 5 Centro de Diagnóstico de Enfermedades Moleculares (CEDEM), Madrid, Spain; Florida Atlantic University, UNITED STATES

## Abstract

The *spf/ash* mouse model of ornithine transcarbamylase (OTC) deficiency, a severe urea cycle disorder, is caused by a mutation (c.386G>A; p.R129H) in the last nucleotide of exon 4 of the *Otc* gene, affecting the 5’ splice site and resulting in partial use of a cryptic splice site 48 bp into the adjacent intron. The equivalent nucleotide change and predicted amino acid change is found in OTC deficient patients. Here we have used liver tissue and minigene assays to dissect the transcriptional profile resulting from the “*spf/ash”* mutation in mice and man. For the mutant mouse, we confirmed liver transcripts corresponding to partial intron 4 retention by the use of the c.386+48 cryptic site and to normally spliced transcripts, with exon 4 always containing the c.386G>A (p.R129H) variant. In contrast, the OTC patient exhibited exon 4 skipping or c.386G>A (p.R129H)-variant exon 4 retention by using the natural or a cryptic splice site at nucleotide position c.386+4. The corresponding OTC tissue enzyme activities were between 3-6% of normal control in mouse and human liver. The use of the cryptic splice sites was reproduced in minigenes carrying murine or human mutant sequences. Some normally spliced transcripts could be detected in minigenes in both cases. Antisense oligonucleotides designed to block the murine cryptic +48 site were used in minigenes in an attempt to redirect splicing to the natural site. The results highlight the relevance of in depth investigations of the molecular mechanisms of splicing mutations and potential therapeutic approaches. Notably, they emphasize the fact that findings in animal models may not be applicable for human patients due to the different genomic context of the mutations.

## Introduction

Ornithine transcarbamylase (OTC) deficiency (OMIM 311250) is the most frequent defect among disorders of the urea cycle, the metabolic pathway that removes waste nitrogen from the body. In the liver, OTC is located in mitochondria of periportal hepatocytes and catalyses the condensation of carbamyl phosphate with ornithine for the formation of citrulline. Since OTC deficiency is X-linked, hemizygous males generally exhibit severe symptoms in the neonatal period while heterozygous females show variable phenotypes depending on the pattern of X inactivation in hepatocytes. However, phenotypic heterogeneity is also observed in males correlating with the severity of the mutations and possibly other genetic and environmental factors [1,2,3].

As in other urea cycle disorders, OTC deficiency presents with hyperammonemia and differential diagnosis is based on a characteristic plasma amino acid profile (elevated glutamine and absent or decreased citrulline and arginine), presence of elevated urine orotic acid and uracil and on the genetic analysis of the *OTC* gene. Patients are at risk of potentially fatal acute hyperammonemia and clinical symptoms are mainly caused by the toxic effects of ammonia in brain, including lethargy, seizures, neurological impairment, brain edema and coma [4]. Standard treatment is based on dietary protein restriction to decrease nitrogen load, use of nitrogen scavengers such as sodium phenylbutyrate and sodium benzoate and provision of an adequate supply of citrulline and arginine. In patients with severe forms, liver transplantation has been proven effective for preventing further hyperammonemic crises [3,4].

To date, more than 440 mutations have been described in the *OTC* gene (HGMD Professional Release 2014.1) and include a majority of missense mutations as well as splicing defects, small deletions or insertions and large deletions [5,6]. However, a high proportion (15%) of patients with a biochemical diagnosis of OTC deficiency do not carry a mutation in the *OTC* gene identifiable by conventional methods [5,7], suggesting that the remaining alleles may correspond to mutations localized in the promoter or deep intronic regions, or are due to locus heterogeneity [8].

Two mouse models currently exist for OTC deficiency, which are useful for ongoing and future research in therapeutical approaches for the management of the disease. The sparse fur (*spf*) mouse carries a missense change (p.H117N) that results in partial (10%) OTC liver activity [9], while the *spf/ash* (abnormal skin and hair) mouse model carries a point mutation (c.386G>A) located in the last nucleotide of exon 4 resulting in the missense change p.R129H, that does not impair enzymatic activity [10]. However, transcript analysis in the *spf/ash* mouse liver revealed the presence of greatly reduced *Otc* mRNA levels (10%) corresponding to a normally spliced product (transcript with the missense p.R129H change) and another product resulting from the use of a cryptic splice site at c.386+48, giving rise to an elongated protein (16 amino acids extra) which is degraded rapidly and is inactive [10]. Presumably, the 90% decrease in transcripts detected as *Otc* mRNA results from other aberrant splicing events possibly causing a frameshift and a premature termination codon and are degraded by the nonsense mediated decay (NMD) mechanism. Overall, hepatic OTC activity in the *spf/ash* mouse was reported by several authors to be 5–10% of wild-type levels [11,12,13], which is attributed to the normally spliced allele with the p.R129H change. This confers a mild phenotype with elevated urinary orotic acid levels but no clinically significant hyperammonemia [10].

Interestingly, several OTC deficient patients carrying the corresponding nucleotide change, c.386G>A have been identified (Table 1). The mutation lies in a CpG dinucleotide explaining the recurrence. As shown in Table 1, the “*spf/ash”* mutation is present in males as well as in asymptomatic and manifesting female patients and is associated both with early and with late onset of the disease. In two Spanish families carrying the mutation, two of the male patients and a clinically manifesting female had normal physical and mental development [14]. OTC activity in jejunal biopsies from female and male patients with this mutation was very low but detectable (1.3–3.5% relative to control values) [14].

**Table 1:** OTC deficient patients with the c.386G>A mutation.

No. of patients	Sex	Onset	Phenotype and outcome	Reference
1	female	late	mild hyperammonemia psychiatric symptoms	[39]
1	male	early	n.d.a.	[40]
6	5 males, 1 female	early/late	3 died, 3 with recurrent crises & normal development	[14]
5	n.d.a.	n.d.a.	n.d.a.	[41]
1	male	neonatal	n.d.a.	[42]
at least 2	1 female/ male (no number given)	”manifesting female”/ late	n.d.a.	[2]
1	female	late	mild hyperammonemia normal neurology	[43]
1	male	late	no hyperammonemia vomiting and confusion leading to diagnosis	this work*
1	male	neonatal	mild course, no recurrent hyperammonemia	this work*
2	females	asymptomaticmothers	normal	this work*
1^#^	male	neonatal	recurrent severe hyperammonemia, neurodevelopmental delay	this work*

n.d.a. no data available

* patients referred to and genotyped at University Children’s Hospital, Zürich.

^#^ Liver tissue from this patient obtained after liver transplantation was used for transcript and enzyme analysis in the present study.

Among the RNA targeting therapies for splicing mutations, one of the most promising experimental approaches today is the use of antisense oligonucleotides (AONs) that act by sterically blocking the access of the spliceosomal components to targeted pre-mRNA regions [15,16]. AONs have been used to prevent intronic pseudoexon inclusion, block cryptic splice sites, induce exon skipping to overcome nonsense or frameshift mutations, promote therapeutically relevant exon inclusion or generation of specific isoforms [17]. The use of AON to block cryptic splice sites activated by mutations can provide a therapeutic approach to recover normal splicing and functional protein. In this sense, the *spf/ash* mouse could represent a natural animal model in which to test antisense therapy for a liver enzyme, taking into account that the missense mutation (p.R129H) resulting from correctly spliced mRNA does not affect mitochondrial import, subunit assembly or enzymatic activity of the OTC enzyme [10]. As mentioned above, human patients carrying the same nucleotide change have been identified. However, although the *Otc* coding sequence has 92% identity to the human sequence, intronic sequences vary to a great extent. In this work, we sought to study the molecular mechanism of the pathogenic nature of the c.386A>G mutation in mice and man, analyzing human and murine liver samples and minigenes. In addition we have investigated the therapeutic potential of AONs designed to block cryptic splice sites.

## Materials and Methods

### RT-PCR analysis in liver samples

Mouse liver samples (wild-type and *spf/ash*) were obtained in accordance with the guidelines and policies of the Veterinary Office of the State of Zurich and Swiss law on animal protection, the Swiss Federal Act on Animal Protection (1978), and the Swiss Animal Protection Ordinance (1981). Animal studies received approval from by the Cantonal Veterinary Office, Zurich, and the Cantonal Committee for Animal Experiments, Zurich, Switzerland (permission for animal experiments Kt ZH130-2014). For this study we used frozen liver samples obtained in a previous study and no animals were specifically sacrificed.

Human liver sample was obtained from a male OTC patient (listed in Table 1) undergoing liver transplantation. Ethical approval for the use of human samples in the study was granted by the Institutional Ethics Committee (reference KEK-ZH-Nr. 2014–0628).

For *in vivo* studies with wild-type or *spf/ash* mice, total RNA was isolated from 20 to 30 mg of mouse liver tissue using QIAmp RNA blood mini kit (Qiagen) according to the manufacturer’s protocol. Random primed cDNA was prepared from 1 μg of total RNA using the Reverse Transcription kit (Promega). PCR amplification of the region from exon 1 to exon 10 of the *Otc*-mRNA was performed using the following primers: forward primer 5’-ATGCTGTCTAATTTGAGGATCCTGC-3’ and reverse primer 5’-AGCACAGGTGAGTAGTCTGTCAGCA-3’. PCR for 40 cycles using PfuUtral II Fusion HS DNA polymerase (Agilent Technologies) was performed at an annealing temperature of 50°C. Amplified products were separated by agarose gel electrophoresis and the products were analyzed by direct sequencing with the same forward and reverse primers after extraction with NucleoSpin Gel and PCR clean-up (Macherey-Nagel).

Human liver samples were immediately stored after biopsies at a temperature of -80°C and investigated using a published protocol with slight modifications [8]. In brief, tissue was disrupted and homogenized using the QIAgen TissueRuptor (QIAGEN GmbH). Extraction of total RNA was performed according to the QIAGEN RNeasy Kit (QIAGEN). Hence, 1 μg of total RNA was used for cDNA synthesis in the presence of PrimeScript II RTase (Takara Clontech). cDNA was amplified with the following primers, generating two overlapping OTC fragments: fragment 1 forward primer: 5’GAAGATGCTGTTTAATCTGAGG, reverse primer: 5’CTGGAGCGTGAGGTAATCAGCC (expected size 580 bp); fragment 2 forward primer: 5’GCAGATGCAGTATTGGCTCG, reverse primer: 5’CCCATACCACGTGTTAGGGATT (expected size 814 bp). In the patient and wild-type control, a full length amplification of the transcript was performed using the forward primer of fragment 1 and the reverse primer of fragment 2 (expected size 1220 bp). For the purpose of sequencing all primers contained M13-tags. PCRs were performed at an annealing temperature of 60 °C.

### OTC activity

OTC activity was determined from mouse and human liver samples in triplicates, following established procedures [18].

### Minigenes

For evaluation of *in vitro* splicing using minigenes, PCR-amplified genomic fragments including *OTC* exon 4 and flanking intronic sequences from human (control fibroblasts) or mouse (C57/BL6) were cloned in the pSPL3 vector (Exon Trapping System, Gibco, BRL). Control fibroblasts were obtained from Coriell Cell Repositories (NJ, USA) and mouse samples from the Animal Core facility at the Centro de Biología Molecular Severo Ochoa. No mice were sacrificed specifically for this study. Ethical approval for the use of human and mouse samples in the study was granted by the institutional Ethics Committee (Universidad Autónoma de Madrid).

The mutations were introduced by site-directed mutagenesis with the Quikchange II site-directed mutagenesis kit (Agilent Technologies). For the minigene assay, 3·10^5^ Hep3B cells grown in 6-well plates were transfected with a total of 1.5 μg of wild-type or mutant minigenes using JetPEI (Polyplus Transfection). At 24h post-transfection, cells were harvested and total RNA purified using Trizol reagent (Invitrogen). RT-PCR was performed with Superscript III First-Strand Synthesis System and FastStart Taq DNA Polymerase (Roche Applied Science) using vector specific primers SD6 and SA2. To select transcripts resulting from the use of the natural 5’ splice site (5’ss) or the cryptic c.386+4 splice site, specific forward primers were designed hybridizing with exon 4 or exon 4 plus the first four intronic nucleotides and exonic vector sequences, respectively (NAT: 5’-ACACCGCTCGACCTGGAGATC-3’ and CRYP: 5’-CGCTCGGTTTACCTGGAGATC-3’). Both primers hybridize with murine and human sequences. In all cases, SA2 was used as antisense primer. Amplified products were separated by agarose gel electrophoresis and the excised bands analyzed by direct sequencing after extraction with QIAEX II Gel Extraction kit (Qiagen). All the experiments were reproduced at least three times.

### In silico analysis

To investigate the effect of the c.386G>A mutation on the splice site strength of mouse and human *OTC* exon 4 and the presence of potential cryptic splice sites nearby, we have performed *in silico* analysis using different softwares: Berkeley Drosophila Genome Project Splice Site Prediction (BDGP, http://www.fruitfly.org/seq_tools/splice.html), Analyzer Splice Tool (AST; http://ibis.tau.ac.il/ssat/SpliceSiteFrame.htm), Maximum Entropy Modelling (MaxEnt; http://genes.mit.edu/burgelab/maxent/Xmaxentscan_scoreseq.html) and Human Splicing Finder (HSF, http://www.umd.be/HSF/).

### Antisense oligonucleotides

A 25-mer morpholino AON designed against murine c.386+48 cryptic splice site was provided by Gene Tools (5’- ATCTTCTCTTTTAAACTAACCCATC-3’). An oligonucleotide hybridizing to an intronic region in an unrelated gene was used as negative control. Hep3B cells were transfected with wild-type or mutant minigenes as described above and 24 h later different concentrations of AON (10–30 μM) were transfected using Endoporter (Gene Tools). Cells were harvested after another 24 h, RNA isolated and RT-PCR analysis performed as described above. The experiments were reproduced at least three times.

## Results

### Effect on splicing of the spf/ash mutation in mouse and human

**Fig 1:**
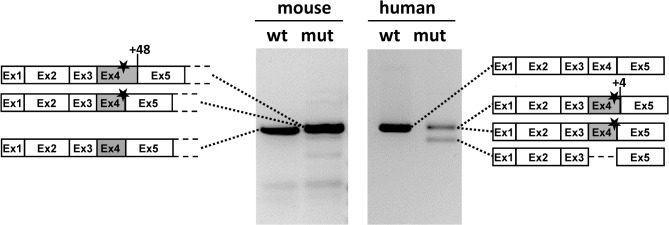
RT-PCR analysis in liver samples from *spf/ash* mouse and from an OTCD patient carrying the analogous c.386G>A mutation. The figure shows the result of amplifying the full length cDNA transcript in mice and a fragment encompassing exons 1 to 5 for the human samples. The schematic drawings on both sides show the identity of the bands which were characterized by sequence analysis (see S1 Fig). The faint bands in the mouse samples could not be sequenced. The star indicates the presence of the c.386G>A mutation. Numbers indicate the position of the cryptic splice sites used.

The effect of the c.386G>A mutation on *OTC* exon 4 splicing was analysed in liver samples from *spf/ash* mice and from a male patient with the equivalent variation. RT-PCR analysis and subsequent DNA sequencing confirmed a splicing defect in both samples (Fig 1 and S1 Fig). For the mutant mouse, the analysis confirmed previously published results [10] as we detected transcripts corresponding to partial intron 4 retention by the use of the c.386+48 cryptic splice site as well as normally spliced transcripts, always containing the c.386G>A (p.R129H) variant. In contrast, in the OTC patient we observed exon 4 skipping or variant (c.386G>A; p.R129H) exon 4 retention by using the natural or a cryptic splice site at nucleotide position c.386+4. The corresponding OTC enzyme activities in liver were 5.6% of normal control (7,2 versus 127,3 μmol/mg/hr) in mouse and 3.7% (0.4 versus 10,9 μmol/mg/hr) in human tissues.

**Fig 2:**
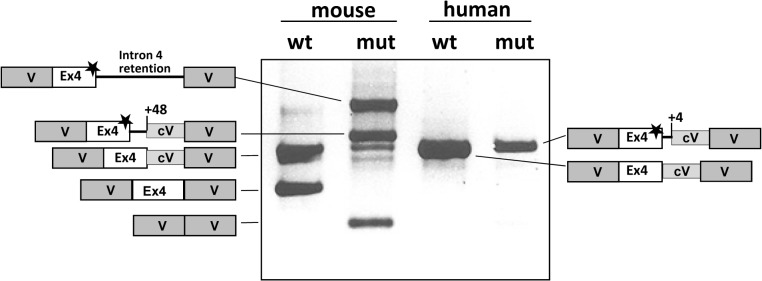
Minigene analysis of the c.386G>A (*spf/ash*) mutation in the murine *Otc* and human *OTC* genes. The gel shows the RT-PCR analysis in Hep3B cells transfected with the wild-type and mutant minigenes carrying the mouse and human genomic sequences (exon 4 and flanking intronic sequences). The schematic drawings on both sides show the identity of the bands which were characterized by sequence analysis. Grey boxes represent vector sequences (V) and white boxes exon 4. The star indicates the presence of the c.386G>A mutation. Numbers indicate the position of the cryptic splice sites used. The box denoted as cV indicates a stretch of vector sequence that is retained in the mRNA due to the use of a cryptic splice acceptor site.

The transcript profile in wild-type and mutant murine and human exon 4 sequences was also analysed *in vitro* using minigenes. As shown in Fig 2, the wild-type murine minigene produced two transcripts both with the correct inclusion of exon 4 and corresponding to either the use of or no use of a cryptic acceptor splice site in the vector. The mutant murine minigene resulted in several aberrant transcripts corresponding to the use of the c.386+48 cryptic splice site, exon skipping and/or retention of entire intron 4. A faint band corresponding in size to the use of the mutant 5’ ss (along with the use of the vector cryptic acceptor splice site) could be detected but its low abundance precluded sequence analysis. In contrast, the mutant human minigene produced only one aberrant transcript corresponding to inclusion of exon 4 plus four adjacent intronic nucleotides that resulted from the use of the c.386+4 cryptic splice site. In the human minigene only the cryptic acceptor site in the vector was used in the splicing process.

Analyzing with different splice prediction programs the murine and human *OTC* exon 4 sequences in its wild-type or mutant (c.386G>A) versions indicated that the natural 5’ splice site lacks a strong splice score, and is not even recognized by the BDGP software (Table 2). The *spf/ash* mutation further decreases the splicing score in both human and murine sequences. In mouse, the cryptic c.386+48 site has a predicted higher splice site strength compared to the natural site. The cryptic splice site c.386+4 is present on both human and murine sequences and has a score comparable to or slightly higher than the mutated 5’ ss, depending on the program used (Table 2).

**Table 2:** *In silico* analysis of *OTC* exon 4 splice sites (ss), including wild-type and mutant (c.386G>A) sequences and cryptic splice sites.

	**MaxEnt**	**BDGP**	**AST**	**HSF**
**Exon 4 *OTC* gene (human)**				
3’ss tttttttattgtag/G	10,3	1	93,6	87,0
Wild-type 5’ss CCG/gtttgt	4,8	0	70,6	81,4
Mutant 5’ss CCA/gtttgt	-1,5	0	58,7	70,8
Cryptic 5’ss (+4) TTT/gtaaat	-2,3	0	63,0	70,2
5’ss (+48)* TGG/ataaat	-	-.	-	-.
**Exon 4 *Otc* gene (mouse)**				
3’ ss ttcttttgttctag/G	11,8	1	94,41	86,3
Wild-type 5’ss TCG/gtttgt	3,5	0	66,6	79,4
Mutant 5’ss TCA/gtttgt	-3,7	0	54,7	68,8
Cryptic 5’ss (+4) TTT/gtaaaa	-6,6	0	57,7	67,9
Cryptic 5’ss (+48) TGG/gttagt	3,1	0,8	76,6	88,1

Higher positive scores indicate a predicted better splice site. The 3’ and 5’ splice acceptor sites are shown, exonic sequences in upper case, intronic sequences in lower case.

* not a cryptic splice site; sequence corresponding to that shown for the cryptic 5’ ss (+48) of the mouse

**Fig 3:**
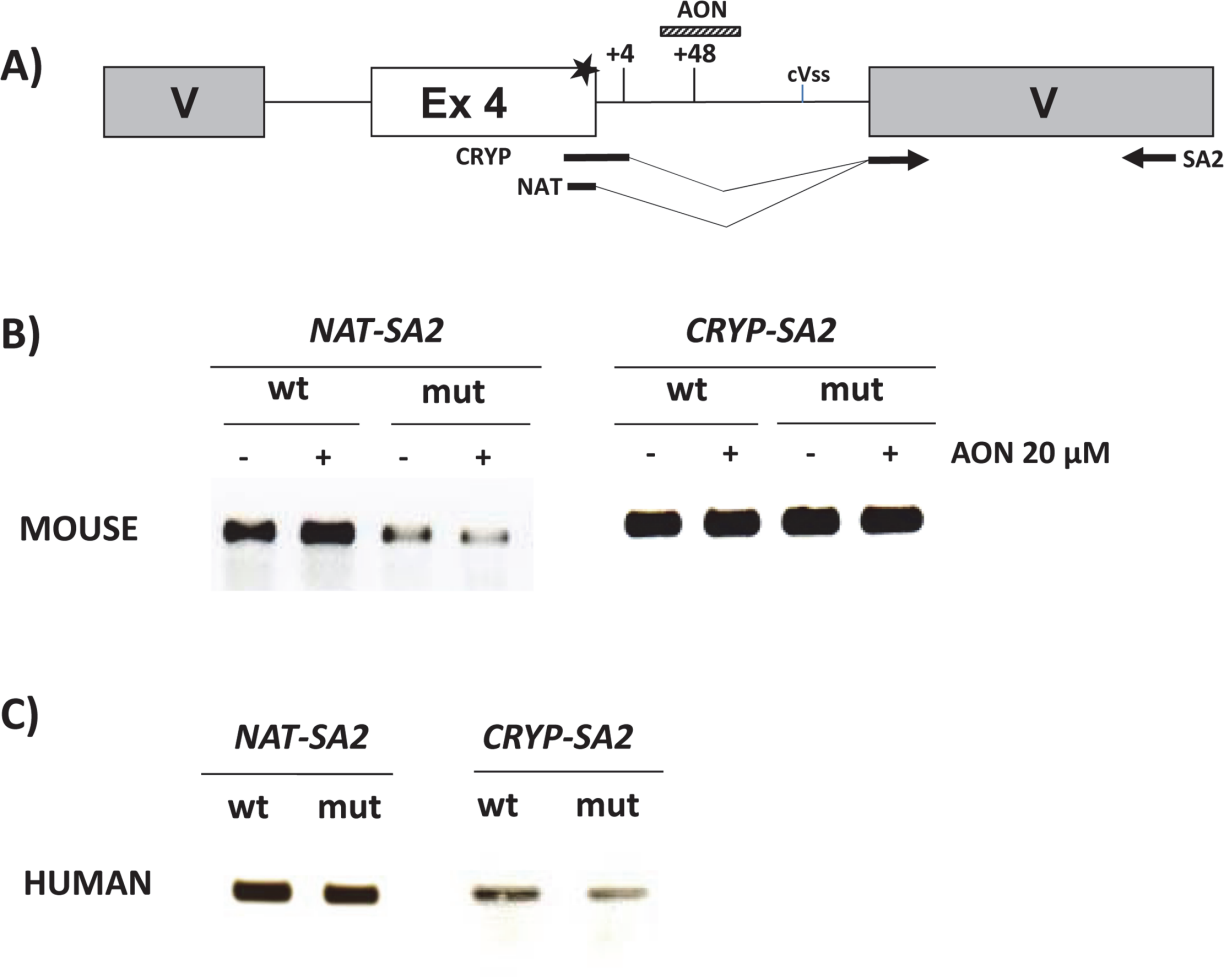
Minigene analysis with specific primers for the natural or cryptic c.386+4 splice sites. A) Schematic drawing of the location of the primers, the murine and human cryptic splice sites and the sequence targeted by the AON. The star indicates the presence of the c.386G>A mutation. cVss: cryptic vector acceptor splice site. B) RT-PCR analysis using the denoted primer pairs obtained for murine wild-type (wt) or mutant (mut) minigenes cotransfected or not with a specific AON targeting the cryptic c.386+48 splice site. C) RT-PCR analysis using the denoted primer pairs obtained in Hep3B cells transfected with human wild-type or mutant minigenes. Sequencing analysis confirmed the identity of the bands and the use of the specific splice site in each case.

In the *spf/ash* mouse and in human liver samples, some normally spliced transcripts (with the c.386G>A change) were detected (Fig 1), so we sought to confirm this in minigenes using a forward primer hybridizing to the junction of exon 4 and the vector exonic sequence (Fig 3A, NAT primer). In addition, the use of the c.386+4 cryptic splice site was also investigated using another specific primer that included the first four nucleotides of intron 4 (Fig 3A, CRYP primer). The normally spliced transcript could be amplified in all cases, although the amount was much lower with the mutant murine minigenes (Fig 3B). This was confirmed by semiquantitative PCR analysis (<30 cyles) (S2 Fig). The results confirm that some amount of normally spliced transcript is produced contributing to the mild phenotype observed in the mouse model and in patients with the mutation. Using the CRYP primer specific for the use of the cryptic c.386+4 splice site, we could also obtain amplified product in wild-type and mutant context, in both the human and murine minigenes, indicating this alternative splice site to be potentially active.

### Using AONs to block the cryptic splice sites activated by the c.386G>A mutation

In the human sequences, the cryptic splice site used (c.386+4) is included in the natural 5’ ss and is involved in base-pair interaction with U1snRNA, thus precluding the possibility of sterically blocking it by AONs. However, in the murine sequences, the antisense approach is feasible for the cryptic c.386+48 site. Having in mind that a mouse model for preclinical development of antisense therapy in metabolic diseases would be highly useful, we investigated the possibility of redirecting transcript processing by a specific splice blocking AON targeted to the c.386+48 cryptic splice site. For this purpose we treated Hep3B cells, previously transfected with wild-type or mutant murine minigenes, with 10–30 μM of AON and analyzed the transcriptional profile.

**Fig 4:**
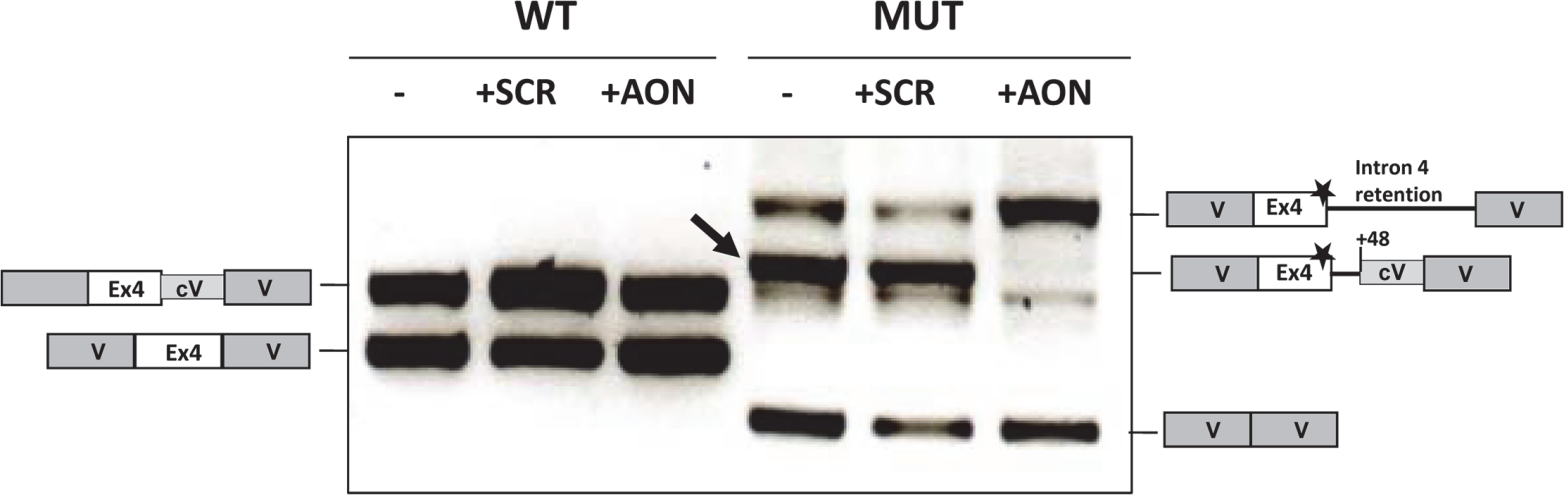
Effect of AON targeting the c.386G>A cryptic splice site on the splicing profile of murine minigenes. Wild-type (wt) and mutant (mut) murine minigenes transfected in Hep3B cells were cotransfected with 20 μM of AON and RT-PCR analysis performed after 24 h. SCR, scrambled oligonucleotide. The schematic drawings on both sides show the identity of the bands which were characterized by sequence analysis. Grey boxes represent vector sequences (V) and white boxes indicate exon 4. Numbers indicate the position of the cryptic splice sites used. The box denoted as cV indicates a stretch of vector DNA that is retained in the mRNA due to the use of a vector cryptic splice site. The star indicates the presence of the c.386G>A mutation. The arrow points to the band corresponding to the use of the cryptic c.386+48 site in the mutant minigenes.

As can be seen in Fig 4, in the presence of AON the band corresponding to the insertion of 48 intronic nucleotides disappeared indicating that the AON efficiently and specifically blocks the use of the cryptic splice site. No effect was visible using a scrambled oligo. Concomitantly, an increase in the band corresponding to intron 4 retention was detected. We did not observe an increase in the band which corresponds by size to the use of the natural splice site or the cryptic c.386+4 site. To corroborate this, we used specific primers NAT and CRYP (Fig 3). As can be seen in Fig 3B, AON treatment did not result in any detectable increase of these specific amplified products.

## Discussion

Splicing defects are estimated to represent up to one third of the disease causing mutations in human genes [19]. Among them, mutations located within the 5’ss are the most common, especially those affecting the invariant GU residues at positions +1 and +2 [20]. Exonic variations even within the 5’ ss are usually interpreted on the basis of the genetic code and without the direct analysis of the patient’s RNA are often not considered to affect splicing. However, in recent years it has become clear that any mutation in the coding sequence may affect pre-mRNA splicing and thus it has been suggested that the “splicing code” overrules the predictions based on the genetic code [21].

In this work we have characterized the c.386G>A change in the *Otc* gene identified initially to be the disease causing mutation in the *spf/ash* mouse model of OTC deficiency and subsequently found in several OTC deficient patients. The mutation is located on the last nucleotide of exon 4 and was classified initially as missense (p.R129H) [22] although subsequent analysis of *Otc* transcripts in the mouse liver revealed that it partially affected the splicing process [10]. Now we have reevaluated the *in vivo* effect on splicing of this change both in murine *spf/ash* and in human OTCD liver samples as well as used *in vitro* experimental approaches based on minigenes.

Exon 4 of the *OTC* gene can be considered a weakly defined exon as its 5’ ss scores low in all splice prediction programs. This means it has more mismatches to the consensus sequence and thus will not be recognized very efficiently by spliceosomal components such as snRNA U1 that binds by base-pair complementarity initiating the splicing process. Based on this *in silico* evaluation it can be hypothesized that exon 4 may be a vulnerable exon prone to splicing defects, which is corroborated by the fact that the c.386G>A mutation caused aberrant splicing in the mouse [10]. Two other mutations affecting the same nucleotide, c.386G>T and c.386G>C, have also been identified in human patients [5]. Both are assumed to affect the splicing process on the basis of their location and the results obtained in the *spf/ash* mouse. In this work we assess the splicing defect of the *spf/ash* mutation in liver from human patients and establish a minigene assay for human and murine exon 4 sequences to reproduce the results and to serve as assay system to test antisense therapeutic approaches. The splicing pattern observed for mutant minigenes mirrored the one *in vivo* relative to the use of cryptic splice sites (c.386+4 in human sequences and c.386+48 in murine sequences). However, exon 4 skipping was observed *in vivo* in human liver while this event was not detected in mouse liver, being detected only for mutant murine minigenes. In this respect, it was shown that the splicing outcome of mutated *NF1* exons 36 and 37 in minigenes depends on the genomic context cloned, indicating that exon definition relies not only in the characteristics of the exons themselves but on the wider genomic environment within which they are located [23]. This also applies to the observed intron 4 retention in mutant mouse minigenes, which may be an artifact related to the short genomic sequence cloned (mouse *Otc* intron 4 is 23,5 Kb long).

The *in vivo* and *in vitro* results correlated well with *in silico* analysis predicting the presence of cryptic splice sites in mouse and human sequences. Previous evidences may aid in understanding the differences observed. In this respect, Roca et al. identified pairwise dependencies between 5’ splice site nucleotides that determine the splicing efficiency [24]. Comparing mouse and human cryptic +4 sites, there is one mismatch difference in mouse (only 4 complementary nucleotides to U1) so a decreased use of the +4 site in mouse compared to humans is expected. The comparison of the results obtained with murine and human minigenes highlights the fact that identical mutations can have a different effect due to the neighboring sequence context, thus making necessary to characterize functionally each change with appropriate experimental approaches. Possibly, inter-species differences in the intronic nucleotides defining auxiliary splicing signals along with local RNA secondary structures and other factors that may affect RNA splicing act together to produce the final splicing profile in each case, as previously discussed [20]. As mentioned before, *in vivo* there may be differences compared to minigenes in the resultant splicing profiles due to the wider natural genomic context. However, *in vitro* analysis in minigenes generally provides an accurate view of the effect of mutations on the splicing process [25,26], extremely useful when transcriptional analysis is not possible.

Based on our previous work on antisense therapy for splicing defects [27], [28] one of our aims was to investigate the feasibility of correcting the *spf/ash* mutation and rescuing OTC expression by the use of AONs. The development of oligonucleotide therapeutics is progressing rapidly, with a large number of disease targets residing in the liver [29]. Antisense therapy with splice switching AONs has several advantages which include the fact that the corrected mRNA is transcribed in its natural context and under its native control and that it is easier to implement than gene therapy. Our hypothesis was that blocking the cryptic splice sites could restore splicing at the natural (albeit mutated) splice site, so a functional protein would be produced, as the missense change p.R129H does not impair enzyme function [10]. However, this was only possible for the murine sequences as in humans the cryptic splice site used is included in the natural splice site.

The results obtained with an AON blocking the c.386G>A indicate that normal splicing cannot be rescued, as other aberrant splicing events are favored. This can be explained by the fact that the mutated splice site has a suboptimal splice score as a consequence of the *spf/ash* mutation. When AONs have been used to block cryptic splice sites, natural splice sites were intact [30], which may be a prerequisite in most cases. Other therapeutic approaches should be investigated in this case, such as the use of adapted U1 snRNA that may compensate for the mutation in the 5’ splice site [31] and drugs that modulate splicing [32,33].

The elucidation of the precise molecular mechanism of the *spf/ash* mutation in mice and man as described in this work offers relevant data for the establishment of genotype-phenotype correlations in OTC deficiency. In the field of splicing defects the relationship between genotype and phenotype is often elusive due to lack of experimental evidence of the molecular mechanism of the mutations. In general, splicing mutations are primarily associated with complete lack of function and severe effects, especially mutations affecting conserved nucleotides GU and AG at 5’ and 3’ ss, respectively. However there are notable exceptions as some mutations affecting nucleotide +1 have been shown to account for a functional protein variant due to activation of a cryptic splice site [34,35]. In addition, many splicing mutations are “leaky” resulting in partial correct splicing that accounts for milder phenotypes [36,37]. Patients with the *spf/ash* mutation retain some residual activity, and can have mild or even no hyperammonemia and normal development (Table 1), indicating a milder effect of the mutation in some patients. In agreement with this, the *spf/ash* mouse also exhibits a milder phenotype [10]. In humans, use of the c.386+4 cryptic splice site would result in an out-of-frame transcript. In mice, the use of the c.386+48 site results in a non-functional elongated protein [10]. Exon skipping would result in an out of frame transcript. The presence of correctly spliced OTC transcripts (coding for the p.R129H variant) was observed *in vitro* and *in vivo* providing an explanation for the not always severe phenotype in mice and man. Interindividual differences in the expression of splicing factors may influence the relative abundance of correctly spliced and aberrant transcripts leading to phenotypic variability observed (Table 1), as has been discussed in other cases for patients with the same genotype [38].

In conclusion, the detailed analysis of the *spf/ash* mutation molecular pathology has allowed establishing the relationship to the phenotype and the investigation of potential targeted therapies. The minigene system may be used to evaluate other point mutations in exon 4 which may affect the splicing process, taking into account that exon 4 may be vulnerable to splicing defects.

## Supporting Information

S1 Fig Sequence analysis of the amplified products obtained after RT-PCR from murine and human liver samples. The chromatograms show the sequencing results of the bands shown in Fig 1 corresponding to RT-PCR analysis form wild-type mouse samples (A), spf/ash mouse samples (B), wild type human liver (C) and liver samples from the OTCD patient, upper band (D) and lower band (E).
(PDF)

S2 Fig Semiquantitative RT-PCR analysis in minigenes using specific primers for the natural or cryptic c.386+4 splice sites. The gel shows the results of RT-PCR analysis (limiting the number of cycles in the PCR to 30) obtained for murine wild-type (wt) or mutant (mut) minigenes cotransfected or not with a specific AON targeting the cryptic c.386+48 splice site, as shown in Fig 3.
(TIF)
