# Correction: Functional Characterization of the *spf/ash* Splicing Variation in OTC Deficiency of Mice and Man

**DOI:** 10.1371/journal.pone.0128506

**Published:** 2015-05-19

**Authors:** Ana Rivera-Barahona, Rocío Sánchez-Alcudia, Hiu Man Viecelli, Veronique Rüfenacht, Belén Pérez, Magdalena Ugarte, Johannes Häberle, Beat Thöny, Lourdes Ruiz Desviat

The following information is missing from the Funding section: This work was supported by Swiss National Science Foundation (grant 310030_153196 to J.H.) and Ministerio de Economia y Competitividad (grant SAF2013-43005-R to LRD). The authors also aknowledge the financial support by the "Rare Disease Initiative Zurich (radiz) program from the University of Zurich".
