# Supplementary material for: Functional Characterization of the spf/ash Splicing Variation in OTC Deficiency of Mice and Man
Source: PLoS One. 2015 Apr 8;10(4):e0122966. doi: 10.1371/journal.pone.0122966 (PMC4390381; doi:10.1371/journal.pone.0122966)

A

Wild-type mouse *Otc* gene sequence (exon 4 - exon 5)

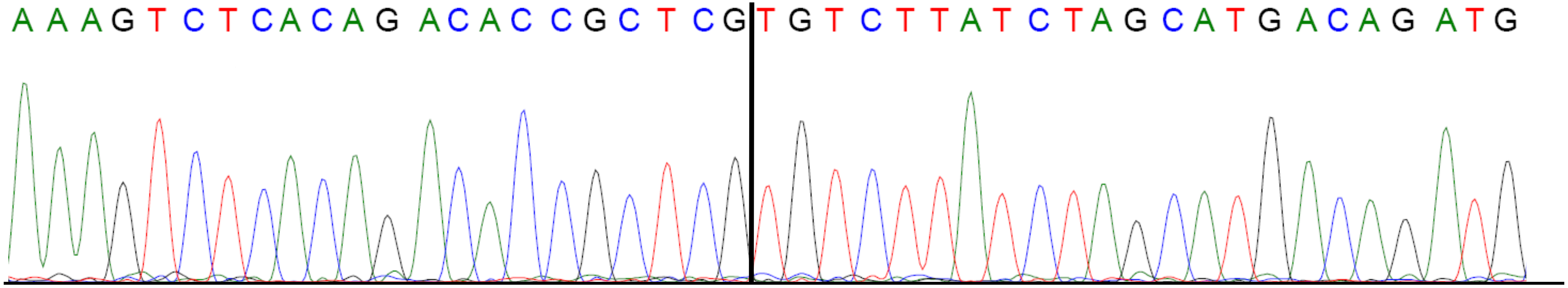

B

Mouse *Otc* gene sequence with the *spf-ash* mutation (exon 4 - intron 4/exon 5)

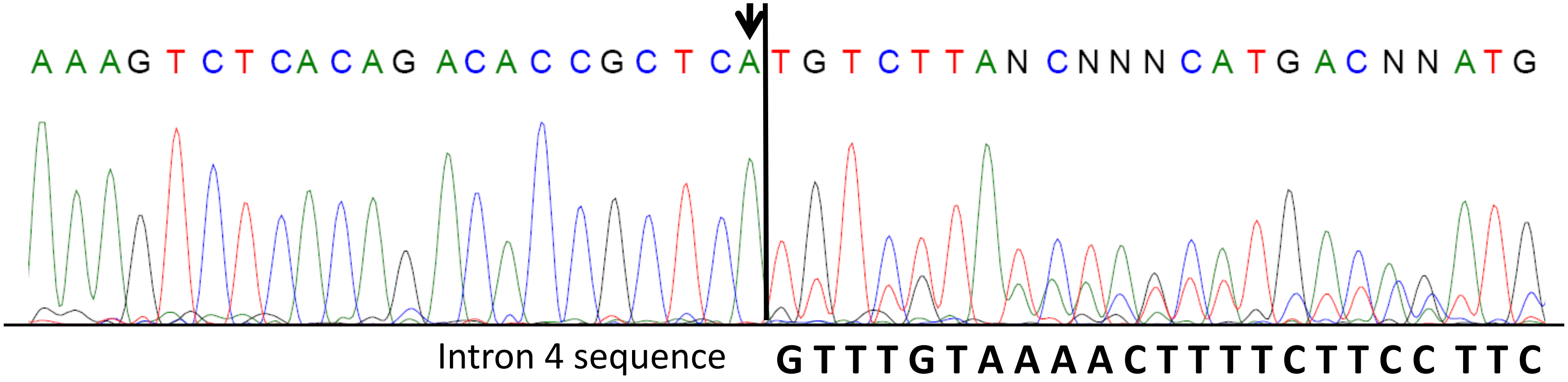

↓ G to A missense mutation

C

Wild-type human *OTC* gene sequence (exon 4 – exon 5)

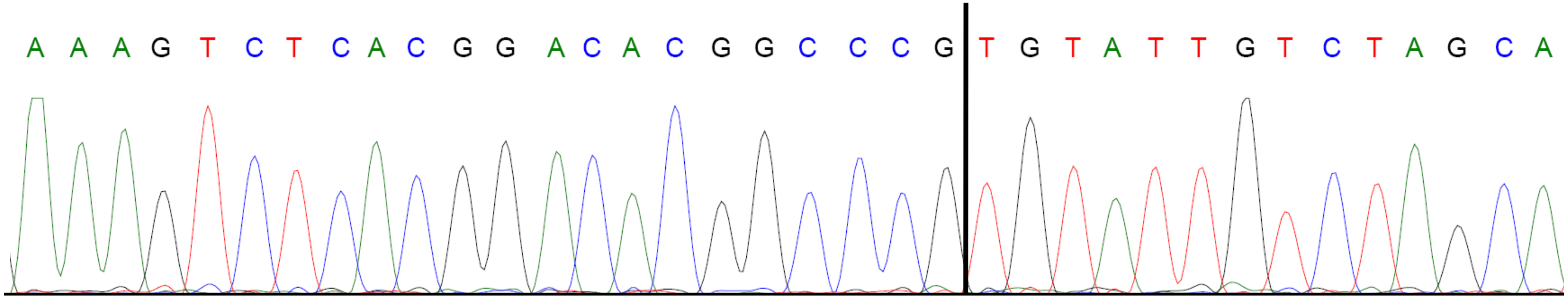

D

Human *OTC* gene sequence with the mutation c.38G>A/p.R129H (exon 3 – exon 5 and exon 4 – intron 4/exon 5)

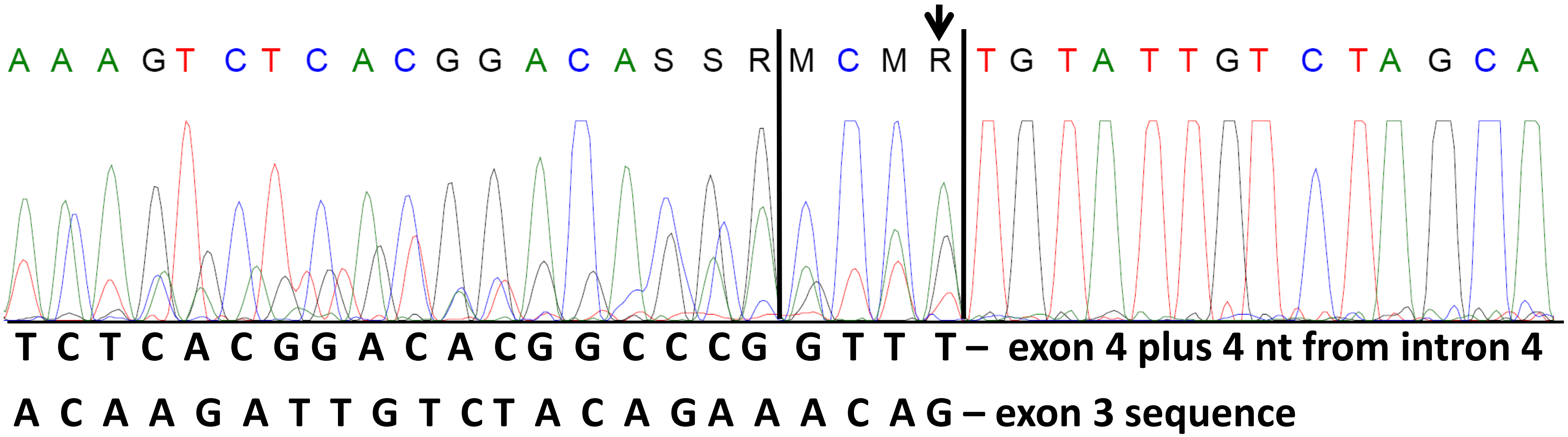

↓ G to A missense mutation

E

Human *OTC* gene sequence with exon 4 skipping (exon 3 – exon 5)

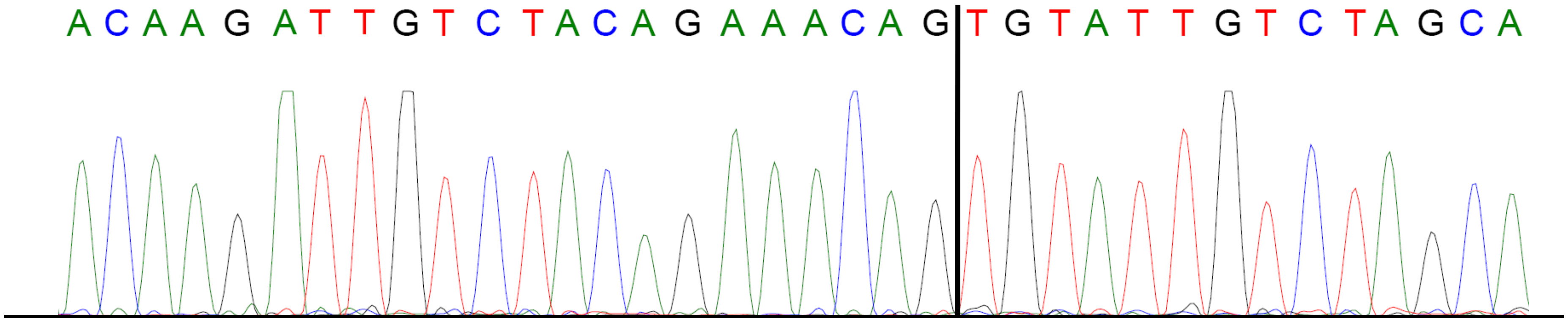

Supplement: S1 Fig — The chromatograms show the sequencing results of the bands shown in Fig 1 corresponding to RT-PCR analysis form wild-type mouse samples (A), spf/ash mouse samples (B), wild type human liver (C) and liver samples from the OTCD patient, upper band (D) and lower band (E). (PDF) [file pone.0122966.s001.pdf]
