# Supplementary figures and images for: Functional Characterization of the spf/ash Splicing Variation in OTC Deficiency of Mice and Man
Source: PLoS One. 2015 Apr 8;10(4):e0122966. doi: 10.1371/journal.pone.0122966 (PMC4390381; doi:10.1371/journal.pone.0122966)

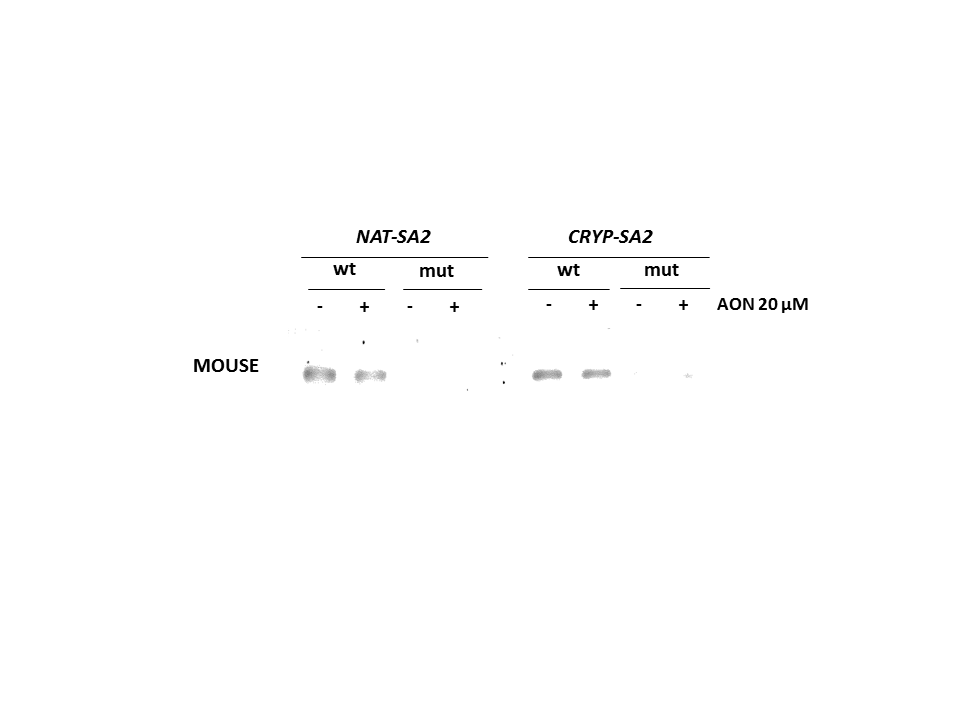

Supplement: S2 Fig — The gel shows the results of RT-PCR analysis (limiting the number of cycles in the PCR to 30) obtained for murine wild-type (wt) or mutant (mut) minigenes cotransfected or not with a specific AON targeting the cryptic c.386+48 splice site, as shown in Fig 3. (TIF) [file pone.0122966.s002.tif]
